# Below versus above Ground Plant Sources of Abscisic Acid (ABA) at the Heart of Tropical Forest Response to Warming

**DOI:** 10.3390/ijms19072023

**Published:** 2018-07-12

**Authors:** Israel de Jesus Sampaio Filho, Kolby Jeremiah Jardine, Rosilena Conceição Azevedo de Oliveira, Bruno Oliva Gimenez, Leticia Oliveira Cobello, Luani Rosa de Oliveira Piva, Luiz Antonio Candido, Niro Higuchi, Jeffrey Quintin Chambers

**Affiliations:** 1 National Institute for Amazon Research (INPA), Ave. Andre Araujo 2936, Campus II, Building LBA, Manaus, AM 69080-97, Brazil; israelmdt@gmail.com (I.d.J.S.F.); bruno.oliva.gimenez@gmail.com (B.O.G.); cobelloleticia@gmail.com (L.O.C.); lcandido@inpa.gov.br (L.A.C.); higuchi.niro@gmail.com (N.H.); jchambers@lbl.gov (J.Q.C.); 2 Climate Science Department, Earth Science Division, Lawrence Berkeley National Laboratory, One Cyclotron Rd., Building 64-241, Berkeley, CA 94720, USA; 3 Federal University of Amazonas, Ave. General Rodrigo Otávio, 1200, Forest Sciences, Manaus, AM 69067-005, Brazil; rosilenaoliveira@yahoo.com.br; 4 Federal University of Paraná (UFPR), Ave. Pref. Lothario Meissner 632, Campus III, Forest Sciences Department, Curitiba, PR 80210-170, Brazil; luani91@gmail.com

**Keywords:** abscisic acid, stomatal conductance, tropical forests, high temperature, drought, isohydric plants, anisohydric plants, isoprenoids, isoprene, monoterpenes

## Abstract

Warming surface temperatures and increasing frequency and duration of widespread droughts threaten the health of natural forests and agricultural crops. High temperatures (HT) and intense droughts can lead to the excessive plant water loss and the accumulation of reactive oxygen species (ROS) resulting in extensive physical and oxidative damage to sensitive plant components including photosynthetic membranes. ROS signaling is tightly integrated with signaling mechanisms of the potent phytohormone abscisic acid (ABA), which stimulates stomatal closure leading to a reduction in transpiration and net photosynthesis, alters hydraulic conductivities, and activates defense gene expression including antioxidant systems. While generally assumed to be produced in roots and transported to shoots following drought stress, recent evidence suggests that a large fraction of plant ABA is produced in leaves via the isoprenoid pathway. Thus, through stomatal regulation and stress signaling which alters water and carbon fluxes, we highlight the fact that ABA lies at the heart of the Carbon-Water-ROS Nexus of plant response to HT and drought stress. We discuss the current state of knowledge of ABA biosynthesis, transport, and degradation and the role of ABA and other isoprenoids in the oxidative stress response. We discuss potential variations in ABA production and stomatal sensitivity among different plant functional types including isohydric/anisohydric and pioneer/climax tree species. We describe experiments that would demonstrate the possibility of a direct energetic and carbon link between leaf ABA biosynthesis and photosynthesis, and discuss the potential for a positive feedback between leaf warming and enhanced ABA production together with reduced stomatal conductance and transpiration. Finally, we propose a new modeling framework to capture these interactions. We conclude by discussing the importance of ABA in diverse tropical ecosystems through increases in the thermotolerance of photosynthesis to drought and heat stress, and the global importance of these mechanisms to carbon and water cycling under climate change scenarios.

## 1. Introduction

### 1.1. Global Increase of Atmospheric CO_2_ and Surface Warming Trends

In January 2015, free troposphere observations by the National Oceanic and Atmospheric Administration (NOAA) in the northern hemisphere revealed that atmospheric carbon dioxide (CO_2_) mixing ratios reached 400 ppm, for the first time in recorded history, and two years later (February 2017), CO_2_ levels had already climbed to 406.42 ppm (http://www.esrl.noaa.gov/). Direct observations of the radiative impact of increasing atmospheric CO_2_ obtained using Atmospheric Emitted Radiance Interferometer (AERI) spectra revealed that the 22 ppm increase in atmospheric CO_2_ between 2000 and 2010 resulted in an increase in CO_2_ surface radiative forcing by +0.2 W·m^−2^ per decade [1]. Also evident in the NOAA and AERI time series are the strong temporal variations due to surface biological processes including photosynthesis and respiration, which in turn affects the surface energy balance. Instead of accumulating in the atmosphere, an estimated half of the current anthropogenic CO_2_ emissions are absorbed by oceans and terrestrial ecosystems [2], demonstrating a large mitigating effect of anthropogenic warming, in part by surface biological processes. In particular, tropical forests, with their rich biodiversity, play a central role in Earth’s climate system by cycling more water and CO_2_ than any other biome [3].

### 1.2. Tropical Forest CO_2_ and Water Fluxes during Warming and Drought Conditions

Tropical forests absorb large amounts of atmospheric CO_2_, accounting for ~34% (42 PgC·year^−1^) [4] of total global terrestrial gross primary production (GPP). Part of this assimilated carbon in the tropics is lost to the atmosphere during autotrophic respiration (R_a_) with the remaining flux (net primary production, NPP) accounting for ~35% of the total global NPP (22 PgC·year^−1^) [5,6]. Most NPP is stored as biomass with tropical forests accounting for 66% (~262 PgC) of the global total [5]; equivalent to ~1.7 times the terrestrial carbon sink since 1850 [7,8]. One of the largest terrestrial carbon sinks on Earth is the Amazon rainforest, with a total stock estimated around 120 PgC, in aboveground biomass and soils [9,10]. In addition, the Amazon cycles a large amount of carbon in the form of CO_2_ with the atmosphere via photosynthesis and respiration with an estimated annual flux of 18 PgC, which exceeds the rate of anthropogenic fossil fuel emissions [11]. However, a high drought sensitivity of this large terrestrial carbon sink has been increasingly documented, including reductions in net primary productivity (NPP), decreases in biomass gains, and increased vegetation mortality during the widespread 2005, 2010, and 2015 Amazonian droughts [2,9,12,13,14]. Moreover, climate models consistently predict warmer conditions in the Amazon basin by the end of the 21st century [15] and a higher frequency (e.g., every 5 years) and intensity of large-scale Amazonian droughts [2,16]. Therefore, climate change factors, including warming trends and droughts threatens the ability of tropical ecosystems to maintain a net carbon sink throughout the 21st century, and consequently mitigate anthropogenic climate effects in the atmosphere. Thus, there is an urgent need to better understand the biochemical and physiological mechanisms underlying forest drought response [17,18], and in particular the combined impacts of high leaf temperatures/light and low moisture availability on net carbon assimilation rates [19,20]. However, the mechanisms by which tropical trees respond and are negatively affected by these factors is an area of intense research. High temperatures and droughts can result in extensive oxidative damage to sensitive plant components such as photosynthetic membranes [21,22]. Understanding how plants respond to oxidative stress is key to being able to predict and perhaps mitigate some of the resulting impacts on tropical forest biodiversity, structure, and function as a globally important net carbon sink.

### 1.3. Plants Hydraulic Strategies in Response to Warming and Water Deficit

Water transport from the soil to the plant to the atmosphere can be viewed as a soil-plant-atmosphere continuum [23] with evaporative water vapor loss to the atmosphere in leaves driven by a water vapor pressure gradient between the sub-stomatal cavities and the atmosphere together with plant water replenishment via root water uptake driven by a gradient between soil and root water potential. Finally, transport of water via the xylem from roots to leaves is driven by a gradient between root and leaf water potential. Thus, precipitation (which influences soil moisture availability to roots) and temperature (which controls the vapor pressure of water) have been described as the main meterological variables influencing interannual diameter increment and tree growth in the Amazon Forest [24].

One the key plant traits known to be involved in drought and high temperature adaptation is the regulation of leaf water potential (Ψ_l_) [25]. Isohydric plants are able to regulate stomatal conductance (g_s_) under high atmospheric demand for water vapor (Vapor Pressure Deficit, VPD) and therefore reduce declines in daily Ψ_l_ and transpiration, thereby reducing the likelihood of hydraulic failure. Hydraulic failure occurs when water tension in the xylem increases, enhancing the risk of xylem embolism, cavitation or collapse, and decrease or complete loss of transpiration [26]. In contrast, anisohydric plants show a reduced ability to regulate g_s_ under high VPD, and consequently can be exposed to high risks of hydraulic failure under drought and high temperatures. Anisohydric plants have been shown to diverge from isohydric plants by having a lower Ψ_l_ while maintaining high g_s_, show a lower sensitivity of g_s_ to decreases in Ψ_l_, and a higher variation in Ψ_l_ along the day [27].

### 1.4. Abscisic Acid (ABA) and Reactive Oxygen Species (ROS) Signaling during Warming and Water Deficit

Studies in the Amazon have found high mid-day leaf temperatures up to 42 °C resulting in large leaf-to-atmosphere water vapor pressure deficits, which drive high leaf transpiration rates and reductions in leaf water potentials [28]. To avoid excessive water loss and potential hydraulic failure, an afternoon reduction in stomatal conductance is often observed, resulting in an afternoon depression of leaf net photosynthesis rates [29,30] and ecosystem NPP [31,32].

One of the earliest processes in plant response to HT and drought stress is the rapid accumulation of the isoprenoid hormone abscisic acid (ABA) stimulating stomatal closure [33] and reactive oxygen species (ROS) that initially function as warning signals that activate defense responses before triggering programmed cell death under excessive ROS accumulation [34]. ABA signaling stimulates stomatal closure leading to reductions in transpiration and net photosynthesis [35], increases in hydraulic conductivities, in part through aquaporin activity [36,37], and activation of defense gene expression including the antioxidant enzymes catalase, ascorbate peroxidase, glutathione reductase, and superoxide dismutase [38] as well as other ABA-induced abiotic stress resistance genes [39]. A recent study using next generation sequencing technology found that exogenous application of ABA to tomato fruit revealed the crucial role of ABA in flavonoids synthesis and regulation of antioxidant systems [40]. The three major components of the ABA signaling network have been described including an ABA receptor, a negative regulator (type 2C protein phosphatase, PP2C), and a positive regulator (SNF1-related protein kinase 2, SnRK2). Together they constitute a double negative regulatory system [41] which has been shown to modify the expression of 10,388 genes in tomato [40]. ABA signaling is intimately linked to ROS signaling. For example, stomatal closure by ABA is mediated by ROS signaling within guard cells [42] and increasing biochemical, genetic, and cell biological evidence points to the emerging view that ROS function as second messengers in ABA signaling [43].

Thus, through stomatal regulation and ABA-ROS stress signaling which alters water and carbon fluxes [44,45], it can be hypothesized that ABA lies at the heart of the Carbon-Water Nexus of plant response to HT and drought stress. A growing body of literature suggests that isoprenoids, including ABA, carotenoids, isoprene, and monoterpenes, play important roles in minimizing ROS accumulation in plants through antioxidant mechanisms including the consumption of excess photosynthetic energy during isoprenoid biosynthesis [46], direct ROS-isoprenoid antioxidant reactions [47,48], and signaling properties of oxidation products [49]. Therefore, ABA plays a central role in plant thermotolerance by increasing hydraulic conductivities and decreasing stomatal conductance to help replenish plant water reserves and mitigate oxidative stress resulting in enhanced cell membrane integrity and continued function carbon assimilation via photosynthesis. Because stomatal closure under HT and drought stress reduces plant uptake flux of atmospheric CO_2_, continued efficient operation of carbon assimilation mechanisms is mediated by enhanced re-assimilation of plant internally produced CO_2_ generated by photorespiration, respiration, and various biosynthetic pathways including the isoprenoid and fatty acid pathways [50].

In contrast, if ROS production overwhelms the scavenging action of the antioxidant system, extensive cellular damage including membrane peroxidation and the reduction of ecosystem net primary productivity (NPP) with a shift from terrestrial sinks to sources of atmospheric CO_2_ [21]. Such a shift in tropical forest carbon balance would eliminate a critical ecosystem service and accelerate global warming.

### 1.5. Biochemical Mechanisms of Isohydric and Anisohydric Strategies

Given its important role in inducing g_s_ reductions, ABA has been implicated in isohydric behavior. When leaves of isohydric angiosperms were exposed to reduced Ψ_l_ by modifying external atmospheric pressure, foliar ABA levels rapidly increased. In contrast, when leaves of anisohydric non-flowering plants were exposed to the same reductions in Ψ_l_, significant increases in foliar ABA were not detected [51]. Therefore, it can be hypothesized that isohydric plants show a higher sensitivity of g_s_ to decreases in Ψ_l_, and a lower variation in Ψ_l_ along the day due to foliar accumulation of ABA. In contrast, anisohydric plants may show a lower sensitivity of g_s_ to decreases in Ψ_l_, and a higher variation in Ψ_l_ along the day due to the lack of a strong foliar ABA accumulation. Thus, leaf turgor loss provides an endogenous signal which appears to trigger ABA accumulation and that the high diversity of tropical tree species and their corresponding ecological niches may differ greatly in the turgor loss thresholds that trigger ABA accumulation. For example in the tropics, the classic Neotropical pioneer genera *Vismia* and *Cecropia* dominate large rainforest disturbance gaps in the Amazon Basin [52] where they help accelerate the regeneration of secondary forests by influencing forest successional pathways [53,54,55]. Their success in secondary forests is related to their ability to maintain high values of stomatal conductance and high corresponding rates of net photosynthesis, transpiration, and growth under conditions of full sunlight, high leaf temperatures, and low nutrient availability, often characteristic of tropical landscapes impacted by natural [52] and human [56] disturbances. In addition to high values of stomatal conductance, relative to climax species in mature forests, pioneer species in disturbed forests often have hydraulic characteristics to facilitate water transport including low height, low wood specific gravity (WSG), and large xylem vessel size [57,58].

A high diversity of hydraulic traits in the Amazon has been observed and associated with the large spatial scale of the Basin (7.5 million km^2^) which has a high environmental heterogeneity and range of plant traits and many hyper-diverse ecosystems with a recent estimate of 6727 tree species across the Basin [59]. Changes in plant hydraulic functional traits are highly associated with local variations in soil type, forest structure, and moisture availability. For example, in the central Amazon, valleys (“baixios”) with predominant sandy soils are vertically close to the water table giving essentially unlimited access to water by roots [60]. In contrast, plateau tree roots may not have direct access to the water table which has been observed at more than 30 m depth [61]. In an effort to minimize water stress during the dry season, plateau trees have been documented to enhance surface water availability during the day by hydraulic redistribution at night where water is moved from moist to dry regions of the soil profile [62]. This leads to the hypothesis that trees in the valleys may be associated with anisohydric stomatal characteristics as they have been documented to have both higher soil water availability and facility of moving water from soils to leaves. One characteristic of isohydric species is the low sensitivity of stomatal conductance to increases in VPD. The higher efficient water transport systems in the valley trees are associated with lower heights [63] and wood specific gravity (WSG), larger xylem vessel diameter, and higher stem cross-sectional sapwood area relative to plateau species [60]. This increased water transport efficiency may come at the expense of hydraulic safety [60]. In contrast, trees associated with well drained plateaus areas, with predominant clay soil content, invest in a wider range of vessel diameters potentially reducing the number of vessels that cavitate, and therefore become non-functional, during severe drought [60]. Likewise, high stomatal sensitivities to increases in VPD and/or decreases in Ψ_l_ may be more common in plateau species, which is a characteristic of isohydric species. Therefore isohydric species may invest more heavily in hydraulic safety at the expense of high rates of net photosynthesis.

Thus, it can be hypothesized that tropical “pioneer species” and trees in the valleys (known as baixio areas in Portuguese), which may exhibit characteristics consistent with anisohydric hydraulic strategies, show a reduced foliar accumulation of ABA due to a reduced sensitivity of ABA production and reductions in g_s_ to decreases in Ψ_l_. Thus, the trees in the plateau show hydraulic traits commonly associated with hydraulic safety at the expense of fast growth rates whereas pioneer trees and those of the valleys show traits commonly associated with fast growth at the expense of reduced hydraulic safety.

## 2. Metabolism of Abscisic Acid (ABA)

**Figure 1:**
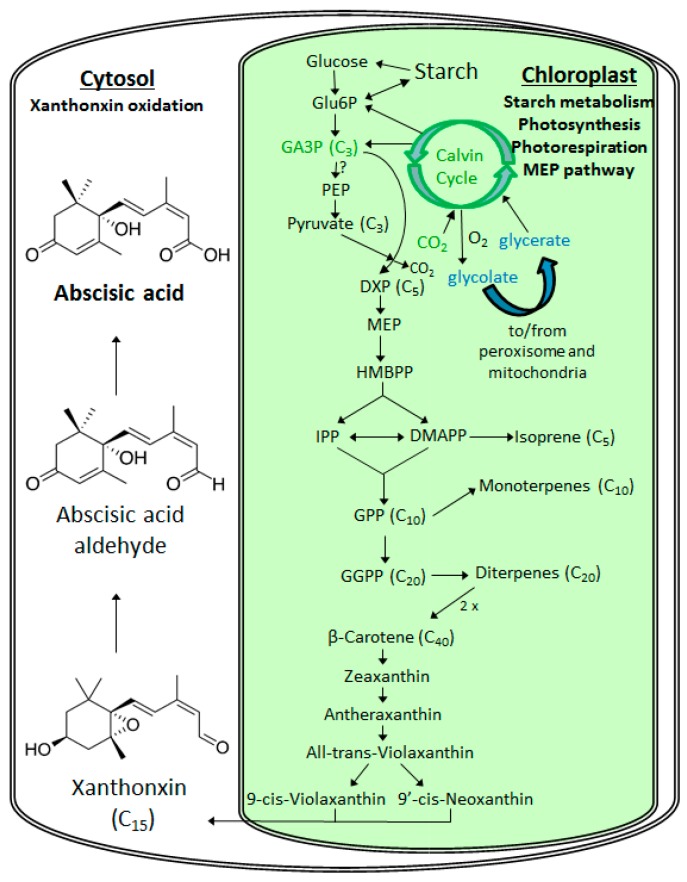
Simplified diagram of the 2-C-methyl-d-erythritol-4-phosphate (MEP) pathway for phytohormone abscisic acid (ABA) biosynthesis with components occurring in the Chloroplast and Cytosol. ABA is derived from C_40_ epoxycarotenoid precursors through an oxidative cleavage reaction in chloroplasts. The C_15_ intermediate xanthoxin is converted to ABA by a two-step reaction via ABA-aldehyde in the cytosol. The green intermediates (CO_2_ and glyceraldehyde-3-phosphate, G3P) represent the substrate and product of photosynthesis in the Calvin Cycle) with blue photorespiratory intermediates (glycolate and glycerate).

Understanding the metabolism of ABA is fundamental to the understanding of its role in the performance of the plant under stress environments including those associated with predictions in future climate including increases in surface temperatures. Abscisic acid biosynthesis begins in the plastids and ends in the cytosol (Figure 1). As a plastidic isoprenoid, ABA derives from C_5_ isoprene units produced in the 2-C-methyl-d-erythritol-4-phosphate (MEP) pathway [64] through the cleavage of C_40_ caratenoids [65]. In this route, ABA is synthesized by cleavage of the C_40_ carotenoid precursor, followed by a two-step conversion of intermediate xanthoxin to ABA via ABA-aldehyde, which will be oxidized to ABA [66]. ABA catabolism is carried out by either oxidative degradation or conjugation with glucose [39]. An emerging view in the literature is that ABA may be produced directly in leaves as the dominant source in plants [67] and here we highlight the possibility that production may occur in photosynthetic plastids (i.e., chloroplasts). Therefore, we hypothesize that the biosynthesis of ABA may have a strong direct connection to photosynthesis for carbon precursors generated as the primary output of the Calvin-Benson cycle reactions (e.g., glyceraldehyde-3-phosphate, GA3P) as well as reducing power (nicotinamide adenine dinucleotide phosphate, NADPH) and energy (adenosine triphosphate, ATP) requirements produced by the light reactions (Figure 1).

### Two Scenarios of ABA Biosynthesis with Distinct Environmental Controls

In the literature, two scenarios related to ABA biosynthesis have been described (Figure 1). The increase in ABA content of root, xylem sap and leaves of drought-stressed plants has been extensively reported [68]. Although the majority of research to-date has focused on ABA production in roots followed by transport to leaves via the transpiration stream (Scenario 1), ABA production is now acknowledged to also occur in leaves (Scenario 2) [69]. Transgenic plants overexpressing key enzymes in the ABA biosynthetic pathway show elevated ABA tissue levels and reduced stomatal conductance [70] with an increased tolerance to drought [71]. Changes in stomatal conductance by guard cells are linked with ABA signaling arriving in the xylem [72], and numerous studies have reported negative correlations between concentrations of ABA in xylem sap and stomatal conductance [73]. However, girdling and decapitation experiments revealed ABA gradients were at least partially determined by local biosynthesis rather than root to leaf transport [74]. As reviewed previously [75], historical studies concluded that the primary plant source of ABA are roots [76] with this biochemical model propagated throughout the literature including its incorporation into widely used leaf gas-exchange models which allow root-derived ABA to be transported to leaves where it impacts stomatal conductance, and therefore fluxes of net photosynthesis and transpiration [77,78]. However, a series of recent studies using foliar application of labeled ABA, reciprocal grafting between ABA biosynthetic mutant and wild-type plants, and stem girdling to block basipetal phloem transport, it was concluded that foliage-derived ABA is readily transported to the roots where it is critical for maintaining normal roots ABA levels and determining root architecture and growth [79,80]. As summarized in the recent review article [75], the results of the two experimental studies concluded that not only is the majority of leaf ABA produced locally in the leaf tissues, leaf-sourced ABA followed by transport to roots dominates root sources of ABA. Thus, the emerging view is that ABA biosynthesis in roots is considered minimal. This conclusion is supported by a previous study which found that leaf response to limited soil water supply was not affected by the capacity to generate ABA in the root, but instead requires ABA biosynthesis and signaling within leaves [81]. Furthermore, these authors concluded that the long-distance communication signal between the roots and leaves is not ABA, but rather a hydraulic signal, which proceeds ABA signaling and stomatal closure.

**Figure 3:**
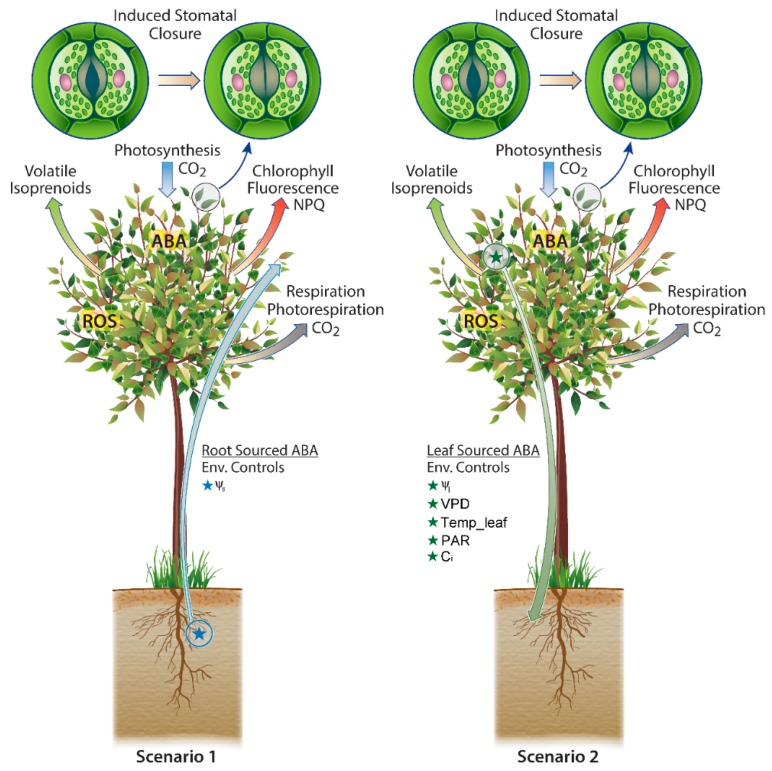
Schematic diagram of two scenarios where ABA mediated plant responses to warming and drought are driven primarily by subsurface processes with environmental controls primarily in soil water potential (Ψ_s_) (Scenario 1) and above ground processes linked with photosynthesis with potential environmental controls including leaf water potential (Ψ_l_), Vapor Pressure Deficit (VPD), leaf temperature (Temp leaf), photosynthetically active radiation (PAR), and leaf internal CO_2_ (Ci) (Scenario 2). In Scenario 1, the blue arrow with a star represents root-derived ABA transported to the leaves. In Scenario 2, the green arrow with a star represents leaf-derived ABA transported to the roots. A list of environmental controls influencing ABA biosynthesis are represented underneath the underlined text Root Sourced ABA and Leaf Sourced ABA.

Isohydric plants are able to rapidly respond to transient water shortages in leaves manifested by decreases in leaf water potential during the late morning to early afternoon by closing their stomata to reduce transpiration leading to a suppression of net photosynthesis [82]. Such a short-term control is difficult to reconcile with the long-distance transport of ABA in trees between roots and leaves and this difficulty is further amplified in the tropics due to low sap velocities (<30 cm·h^−1^) and large tree heights (up to 45 m). Using real-time observations, we present new theoretical estimates of transport times of ABA between roots and the upper canopy from 4 trees in an undisturbed mature tropical forest in the central Amazon during a 12-day dry season period (5–21 May 2015, Figure 2). The results show that for tree heights of 19.8 to 31.0 m, the mean daily sap velocity ranged between to is 0.4 to 1.4 m/day with transport from roots to canopy between 22 and 49 days. These extremely long transport times make the scenario of fast stomatal regulation through root to canopy transport of root-derived ABA unlikely, but support instead the scenario of a direct leaf source of ABA (Figure 3).

Therefore, these two scenarios (not mutually exclusive) describe plant sources of ABA and suggest distinct environmental controls (Figure 3). In the first scenario (Scenario 1), ABA biosynthesis is carried out in the roots and transported to the leaves via the transpiration stream with ABA acting as a whole-plant messenger of low soil water potential and a leaf signal that the plant needs to save water by reducing g_s_. In this classic scenario, root ABA biosynthesis is stimulated by a decrease in soil water potential. In the second scenario (Scenario 2), ABA is produced directly in the leaves in response to a number of physiological and environmental variables including leaf water potential, and VPD. In addition, it can be hypothesized that, due to the tight connection between the Calvin cycle and the MEP pathway (Figure 1), variables influencing photosynthesis including leaf temperature, photosynthetically active radiation (PAR) and leaf internal concentrations of CO_2_ may also affect leaf ABA production. In addition, variables influencing leaf water status including leaf to atmosphere vapor pressure deficit (VPD) and leaf water potential are also expected to influence leaf ABA production.

**Figure 4:**
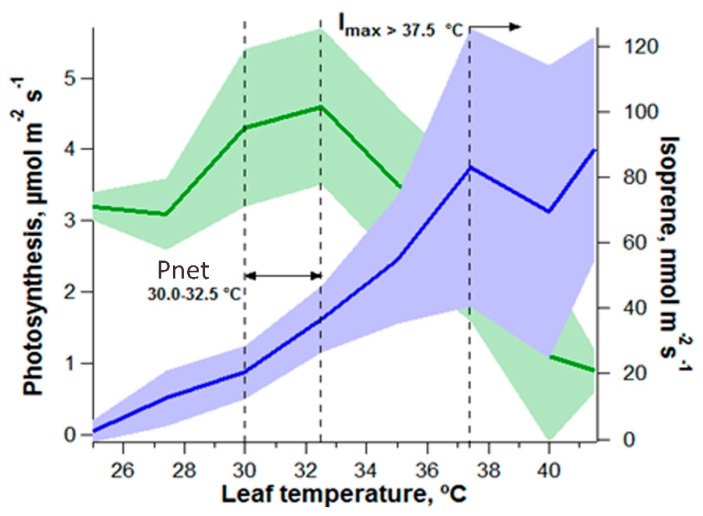
Uncoupling of P_net_ (green) from isoprene emissions (blue) at elevated leaf temperatures as modified from [83] (www.plantphysiol.org, “Copyright American Society of Plant Biologists”). Similar uncoupling of P_net_ and monoterpene emissions at elevated leaf temperatures has also been observed [14]. Vertical dashed line represent optimum temperatures for leaf P_net_ and isoprene emissions (I_max_).

Consistent with the second scenario, in response to high temperature stress, a large number of tropical plants synthesizes a number of secondary defense metabolites via the isoprenoid pathway in chloroplasts (e.g., isoprene and monoterpenes), some of which have sufficient vapor pressures to be directly emitted into the atmosphere at high rates as volatile organic compounds (VOCs). Isoprene and monoterpene emissions generally account for 1–2% of net photosynthesis at leaf temperatures below the optimum for photosynthesis, but 10% or higher at temperatures above this optimum. A large fraction of tropical tree species emit isoprene and/or monoterpene to the atmosphere at high rates. ^13^CO_2_ labeling experiments have shown that these volatile isoprenoid emissions derive from recently assimilated carbon rather than from stored reserves [14,45,83]. Moreover, net photosynthesis (P_net_) and g_s_ generally shows a leaf temperature optimum between 31 and 33 °C [84], while emissions of isoprene and monoterpenes continue to increase up to 40 °C or beyond [14,83]. Observations at the leaf and ecosystem scales in the central Amazon demonstrate the highest isoprenoid emission fluxes during the hottest period of the day (13:00–14:00) when stomatal conductance is reduced [47,85,86,87] (Figure 4). Therefore, as has been shown for isoprene and monoterpenes with de novo biosynthesis linked to photosynthesis for carbon and energy via the MEP pathway, it can be hypothesized that the rates of ABA production may continue to increase with leaf temperature, giving rise to a positive feedback of stomatal closure, the minimization of water loss, and the prevention of hydraulic failure and mortality.

## 3. Modeling of Leaf and Canopy Conductance to Water and CO_2_ in a Changing World

The mechanistic representation of the main source(s) of ABA production in plants into physiological and global models are extremely important for the accurate simulation of the response of different Plant Functional Types (PFTs) widely used in Dynamic Global Vegetation Models (DGVMs) and their responses to climate variables including temperature and moisture availability. For example, approaches have been described that represent the impact of heritable traits on stress tolerance [88]. These efforts will improve the representation of forest structure and function including soil-plant-atmosphere exchange fluxes of H_2_O and CO_2_, which are critical to improve fully coupled Earth System Models (ESMs) aiming to quantify the interactions and feedbacks between terrestrial vegetation and climate. Towards this goal, the root sourced ABA biosynthesis Scenario 1 (Figure 3) has infiltrated the literature, forming the basis for widely used gas-exchange models of various complexity with considerations to include them in large scale land surface models [78,89,90,91,92]. However, with new experimental results demonstrating that the principle plant source of ABA is local production in leaves [67,75,79,80], a new modeling framework based on a Scenario 2 is required (Figure 3).

**Figure 5:**
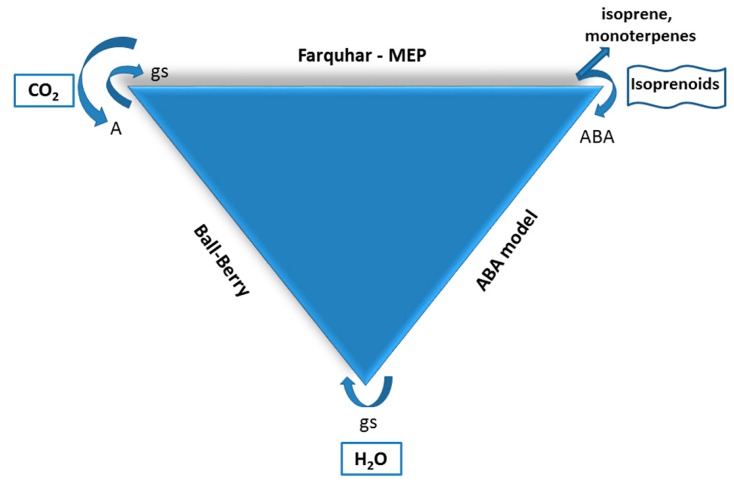
Simplified diagram showing proposed model interactions between a Ball–Berry (stomatal conductance), Farquhar–MEP (photosynthesis-isoprenoid), and isoprenoid emissions and ABA (stomatal conductance) models.

Many ESMs employ the empirical Ball–Berry type models which predicts stomatal conductance based on net photosynthesis rates and environmental conditions including relative humidity and CO_2_ concentrations at the leaf surface [93]. Net photosynthesis rates can be calculated by a Farquhar-Berry type model using stomatal conductance (to derive leaf internal CO_2_) and environmental variables (light, temperature, CO_2_) as input together with kinetic properties of the Ribulose-1,5-bisphosphate carboxylase/oxygenase enzyme (V_cmax_), electron transport (J_max_), and triosphosphate utilization [94]. Thus, sequential operation of the Ball–Berry–Farquhar models in ESMs enables predictions of the response of plant physiological variables including stomatal conductance, transpiration, and net photosynthesis to changes in environmental variables including light, temperature, CO_2_ concentrations, relative humidity, and soil moisture [95]. Here, we propose the development of a photosynthetic energy-linked isoprenoid component, which produces ABA locally within leaves. The integration with an ABA-stomatal conductance model with a conventional Ball-Berry model may lead to improved predictions of stomatal conductance, especially if parameterized across different PFTs including isohydric and anisohydric plants. Thus, by integrating these models, a combined mechanistic representation of environmental, biochemical, and physiological controls over stomatal conductance could be achieved (Figure 5).

## 4. Future Experiments and Studies

### 4.1. Demonstration of Recent Photosynthesis in Leaves as a Principal Carbon Source for ABA

A GC-MS based method [96] could be used with ^13^CO_2_-labeling to provide the first demonstration of photosynthetic carbon sources for ABA. Alternatively, Liquid Chromatography-Mass Spectrometry (LC-MS) could allow for the simultaneous quantification and isotopic analysis of ABA, carotenoids, and other low molecular weight antioxidants and their oxidation products [97]. Thus, ^13^C-isotopic analysis of leaf ABA during ^13^CO_2_-labeling experiments would enable the first estimate of the relative importance of local photosynthetic ABA production versus transported root-derived ABA.

**Figure 6:**
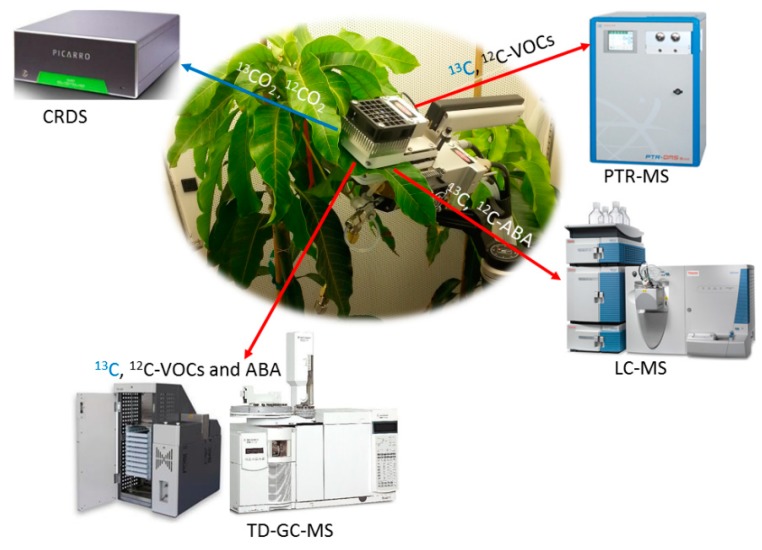
Example of a potential experimental instrumentation configuration for the quantification of ^13^C-labeling analysis of volatile isoprenoids (isoprene and monoterpenes) and non-volatile isoprenoids (e.g., ABA and carotenoids) during photosynthesis under a ^13^CO_2_ atmosphere. The blue arrow represents stable carbon isotope analysis of CO_2_ by laser spectroscopy and the red arrows represents stable carbon isotope analyses of ABA and volatile organic compounds (VOCs) by mass spectrometry.

Novel stable carbon isotope techniques that integrate leaf gas-exchange systems with advanced analytics including Gas Chromatography-Mass Spectrometry (GC-MS), Proton Transfer Reaction-Mass Spectrometry (PTR-MS), and Cavity Ringdown Spectrometry (CRDS) could be applied [83]. This configuration allows for the simultaneous quantification of photosynthesis via ^13^CO_2_ uptake and ^13^C-labeling analysis of volatile (isoprene and monoterpenes) together with offline analysis of ^13^C-ABA labeling using GC-MS and/or LC-MS (Figure 6).

### 4.2. Leaf ABA Biosynthesis as a Function of Leaf Temperature (VPD Constant) and VPD (Leaf Temperature Constant)

Due to their influence over physical (e.g., transpiration and leaf water potential) and biochemical (enzyme activity) leaf properties, the influences of leaf temperature and VPD on leaf ABA biosynthesis/concentrations and g_s_ should be explored independently. However, changes in leaf temperature are often associated with changes in VPD. To decouple these effects on g_s_, leaf temperature g_s_ response curves could be carried out under constant VPD (by also varying relative humidity). In addition, leaf VPD g_s_ response curves could be carried out under constant leaf temperature by only varying relative humidity.

### 4.3. Evaluating the Role of ABA Biosynthesis on Stomatal Control in Distinct Plant Functional Types

Understanding the biochemical mechanisms underlying the different physiological strategies of isohydric and anisohydric stomatal behavior is important for quantifying carbon and water fluxes in terrestrial ecosystems. This is also essential for predicting which species succumb to future climate warming and drought and which species are resistant and survive. Given the potential role of leaf ABA production in regulating g_s_ and activating defenses including antioxidants systems, its role in isohydric/anisohydric and pioneer/climax stomatal behavior in tropical forests deserves attention. For example, new observations of diurnal leaf water potential, g_s_, transpiration, net photosynthesis, and leaf ABA concentrations are needed in the topographic gradient from the valleys to plateaus in response to changing environmental variables including solar radiation, leaf temperature, VPD, ambient CO_2_ concentrations, and soil water content. Similar observations are suggested in disturbed secondary forests dominated by fast-growing pioneer species. While many species only exist in the valley or the plateau, there are many generalist species that occur in both the plateaus and valleys including *Eschweilera coriaceae*, *Protium hebetatum*, *Swartzia tomentifera*, *Gustavia hexapetala* and *Pseudolmedia laevis* [98]. Although the ishohydric/anisohidric behaviors of these generalist species are unclear, it could hypothesized that they demonstrate high phenotypic plasticity of functional hydraulic traits. For example, functional plasticity in the hydraulic architecture and specific leaf area (SLA) has been observed in a perennial herb in response to changes in water availability [99]. Finally, little is known about the role of seasonal variations and leaf phenology on isohydric/anysohydric stomatal behavior in tropical forests and the role of ABA in these interactions.

### 4.4. Quantification Tissue Concentration of ABA, ROS, Antioxidant Capacity, Membrane Peroxidation Biomarkers

In order to evaluate the interactions between ABA and ROS signaling under HT and drought stress in the tropics and their roles in membrane stability and thermotolerance of photosynthesis, a suite of leaf metabolites are recommended to be simultaneously quantified. This includes quantification of ABA, ROS, antioxidant capacity, and membrane peroxidation biomarkers. Experimental kits for the quantification of these metabolites are available based on colorimetric and/or fluorescence detection such as the Enzyme-Linked Immuno Sorbent Assay (ELISA) for ABA [100].

## 5. Conclusions

Under drought and HT stress, the phytohormone ABA has been well documented to induce stomatal closure leading to a reduction in transpiration and net photosynthesis, increase plant hydraulic conductivities, and activates defense gene expression including antioxidant systems. Therefore, ABA lies at the heart of the Carbon-Water-ROS Nexus of plant response to environment extremes and may be a critical plant endogenous factor that integrates hydraulics, carbon and energy metabolism, and defense mechanisms with environmental variables including moisture availability and temperature. Until recently, nearly all plant ABA experimental observations and models of ABA production, stomatal conductance, and gas exchange assumed a root source as the principal source of ABA in plants (Figure 1: Scenario 1). Here we show that the theoretical ABA transport time between the roots and main canopy leaves in the central Amazon is too long (>3 weeks) to account for rapid changes in g_s_ throughout the day (e.g., mid-day suppression of g_s_ associated with high VPD). This is consistent with recent experimental evidence that suggests a leaf source as the principal source of ABA in plants (Figure 1: Scenario 2). As tropical leaf emissions of isoprene and monoterpenes derive from recent photosynthesis via the same biochemical pathway as ABA (MEP), the possibility of a direct energetic and carbon link between leaf ABA biosynthesis and photosynthesis exists. This possibility suggests the potential for a positive feedback between leaf warming and enhanced ABA production together with reduced stomatal conductance and transpiration. Moreover, variations in stomatal sensitivities to increases in VPD and decreases in Ψ_l_ across diverse hydraulic functional traits maybe partially attributed to variations in ABA biosynthesis sensitivities to VPD and Ψ_l_. Thus, species-specific variations in ABA biosynthesis sensitivities to VPD and Ψ_l_, may help explain isohydric stomatal behavior in Amazon forest plateaus and anisohydric stomatal behavior in valleys and secondary forests. Given the predictions of increasing mean surface temperatures and frequency and duration of widespread droughts in the tropics, an accurate representation of stomatal conductance behavior in ESMs is critical for predicting future carbon and water fluxes between terrestrial ecosystems and the atmosphere. For example, a reduced stomatal sensitivity to VPD in valley ecosystems relative to plateau ecosystems may potentially buffer overall decreases in ecosystem NPP during HT stress. This knowledge is also essential for predicting which species succumb to future climate warming and drought and which species are resistant and thrive. If a photosynthetic source of ABA is verified, an integrated gas exchange model could be developed linking photosynthesis and ABA production to stomatal conductance. Such an integrated model could be incorporated into modern ESMs to potentially improve predictions of the interactions and feedbacks between terrestrial ecosystems and the atmosphere under a changing climate.

## Figures and Tables

**Figure 2:**
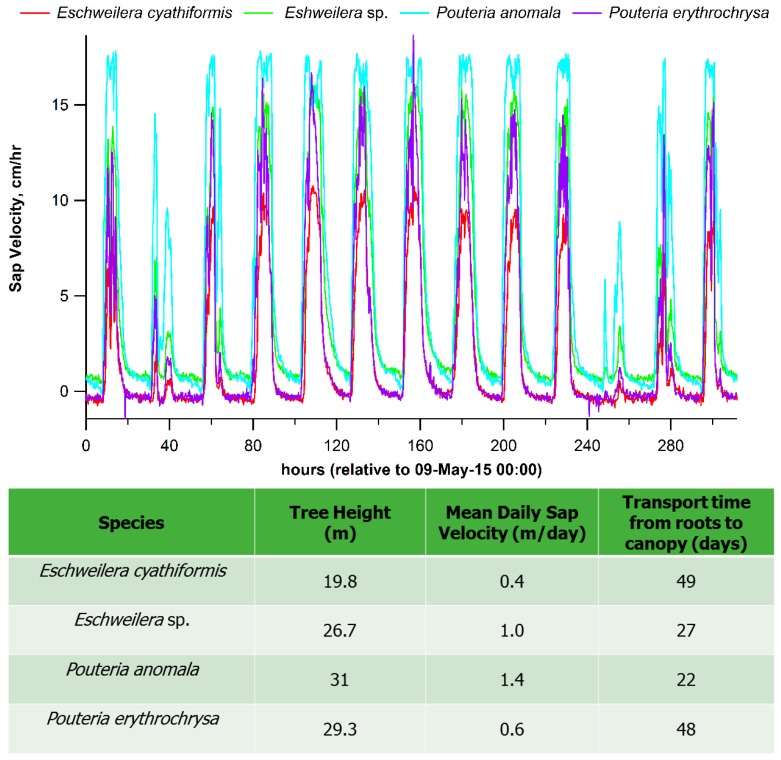
Real-time sap velocities of 4 trees in an undisturbed mature tropical forest in the central Amazon during a 12-day dry season period (5–21 May 2015). Also shown in the table below are calculated transport times required for root-derived ABA to reach the top of the canopy via the transpiration stream.
